# A scoping review of educational practices in caesarean section training

**DOI:** 10.1186/s12909-026-09495-y

**Published:** 2026-05-28

**Authors:** Liesl de Waard, Elize Archer, Kathryn Chu, Pauli van Heerden, GS Gebhardt

**Affiliations:** 1 Department of Obstetrics and Gynaecology, Stellenbosch University, Fransie Van Zijl Drive, Cape Town, 7500 South Africa; 2 Department of Health Professions Education, Stellenbosch University, Cape Town, South Africa; 3 Centre for Global Surgery, Stellenbosch University, Cape Town, South Africa

**Keywords:** Caesarean Section, Training, Assessment, Learning curve, Competence

## Abstract

**Background:**

Caesarean Section (CS) is the most frequently performed abdominal surgery. Procedure-related mortality following CS is up to 100 times higher in Low- and Middle-Income Countries (LMICs) compared to High-Income Countries (HICs) when comparing outcomes between countries classified by the World Bank’s income classification. In LMICs, CS are often conducted by junior doctors with limited supervision, highlighting the need for effective training strategies. This scoping review aimed to systematically identify, describe and map existing educational strategies for training novice surgeons in CS, to identify approaches that may be applicable in LMICs settings.

**Methods:**

This scoping review was conducted according to the guidelines from the Joanna Briggs Institute. Studies describing educational interventions, training strategies or assessment approaches for CS were included, all study designs and data sources were considered. Published literature between 2001 and June 2025 was searched using terms and synonyms related to caesarean section, assessment, training and education. Databases PubMed/Medline, Epistemonikos, EMBASE, ScienceDirect, Web of Science/Scopus, and ERIC/EBSCHOST were searched. Data were extracted using a predefined charting database and synthesis was conducted using a hybrid deductive-inductive approach. Studies were classified according to the World Bank’s 2022–2023 country income levels according to the country in which the study was conducted. Studies were categorised by teaching modality, assessment and learning outcomes, the learning curve and resource requirements. The Kirkpatrick model was applied to evaluate training outcomes.

**Main results:**

A total of 45 data sources were included, 31 (69%) were conducted in HICs, and 14 (31%) in LMICs. Simulation-based training was the most common teaching strategy (53%) and was often combined with video or classroom-based training. When outcomes were assessed, most (75%) reported improvements in knowledge, skills or confidence (Kirkpatrick levels 1–2); few assessed behavioural or clinical outcomes (Kirkpatrick levels 3–4). Improvements in clinical outcomes were mainly observed in large-scale emergency obstetric training programs. Learning curve analyses indicated that operative time and blood loss appear to plateau after 15–30 cases.

**Conclusions:**

Published research on educational strategies for CS is limited, particularly in LMIC settings, and few have been assessed for impact beyond participant-level outcomes. Promising approaches include blended learning, team-based training, and simulations. There is an urgent need for affordable, reproducible training strategies for LMICs, accompanied by an evaluation framework that assesses the impact on clinical outcomes.

**Supplementary Information:**

The online version contains supplementary material available at 10.1186/s12909-026-09495-y.

## Background

Caesarean section (CS) is the most commonly performed abdominal surgical procedure worldwide, with an estimated annual caseload exceeding 33 million procedures [1]. There are concerning differences in outcomes and complications when comparing CS in Low- and Middle-Income countries (LMICs), as defined by the World Bank income classification, to High Income Countries (HICs), with procedure-related mortality up to 100 times higher in LMICs [2, 3]. While several system and patient-related factors contribute to these outcomes, operator proficiency and surgical training key determinants of the quality and safety of CS, particularly in LMICs, where junior doctors are often required to perform the surgery with limited supervision [2, 4–6].

Surgical training has traditionally followed an apprenticeship model, where novice surgeons assist the trainer (principal surgeon) and gradually take on more responsibility until deemed competent to operate independently and train others [7]. However, the ^“^see one, do one, teach one” approach is now considered inadequate [8]. Advancements in medical education, increasing demands for surgical efficiency, medicolegal concerns, theatre time constraints, and growing surgical complexity all challenge this model [9]. A key ethical concern of the traditional apprenticeship model is that trainees acquire skills while operating on patients, potentially compromising safety [10]. Simulators are increasingly used in surgical training, offering a low-risk environment for skill development and assessment [10]. Sultana et al. conducted a systematic review of simulation-based training for CS and identified only 19 studies. They concluded that although simulation-based training may improve both technical and non-technical skills, there is a lack of evidence on its clinical and educational effectiveness [11].

Zetner et al. (2019) and Madsen et al. (2013) reviewed educational practices and strategies for CS, including simulation, classroom-based learning, clinical training, e-learning, videos, and competency assessment [12, 13]. Both highlighted the limited available data and the need for further research including data on competency assessment. None of these reviews included specific recommendations for LMICs, however the authors noted that low-cost simulators may serve as viable alternatives to more expensive models.

Competency assessment plays a crucial role in surgical training by providing feedback opportunities, tracking progress, and ensuring patient safety before a surgeon operates independently [14]. Logbooks are commonly used; however, they quantify experience rather than competence, and do not capture the quality of performance [14]. Structured assessments of technical skills for specific tasks, as well as global rating scales, have proven valuable for both formative and summative assessment and are increasingly utilised across various surgical disciplines, however the extent to which these practices are used for CS training is not well documented [8, 15, 16].

This scoping review aimed to identify and map existing strategies in CS training. Specifically we sought to describe how training programmes are structured (in terms of duration, curriculum, and competency assessment) as well as to identify implementation strategies or barriers. Where reported, measured outcomes were evaluated to assess skills acquisition, retention and improvements in teamwork as well as clinical outcomes (e.g. mortality or morbidity) related to the raining interventions. Identified data were also appraised for applicability to a LMICs setting.

### Objectives

The objective of this scoping review was to systematically identify and map educational strategies used in training clinicians to perform CS in both HICs and LMICs. Training programmes were characterised according to key features of educational interventions, including modality (simulation-based, intraoperative teaching, structured training), target audience, implementation strategies and reported outcomes. The review further aimed to highlight approaches that may be applicable or adaptable to resource-constrained environments.

## Methods

This scoping review was conducted in accordance with the methodological framework established by the Joanna Briggs Institute [17]. The Preferred Reporting Items for Systematic Reviews and Meta-Analyses Extension for Scoping Reviews (PRISMA-ScR) was used to guide the reporting of the study and is available as Supplementary Table S1, with Covidence software used to facilitate these steps [18, 19]. Thematic synthesis was conducted using a hybrid deductive–inductive approach, employing categories identified from the literature while concurrently accommodating new themes emerging during data extraction and analysis [20]. An initial coding framework was developed where pre-existing (a priori) themes were derived from the literature review aligned with the objectives of the study (the deductive phase). This was iteratively expanded to incorporate additional themes generated directly from the data during analysis (in the inductive phase). The content analysis involved the first author (LDW) familiarising herself with the data, after which interpretations were reviewed by the other authors to enhance rigour [21]. Similar concepts were grouped to form codes, which were subsequently refined into themes and sub-themes. The deductive and inductive components were then integrated, allowing the themes to be combined and mutually inform one another.

### Eligibility criteria

We included peer-reviewed studies that focused on CS training, educational and assessment practices, and innovative approaches for training novice surgeons. Additional sources such as academic dissertations, conference proceedings, and poster abstracts were also reviewed for inclusion. The search strategy was guided by the Joanna Briggs Institute’s Participant Concept Context (PCC) framework [17]. The target population consisted of junior doctors learning to perform CS. The concept was centred on training methods for CS (e.g., simulation-based training, e-learning, multidisciplinary drills, or specific skills training). No geographical restrictions were applied. The inclusion and exclusion criteria are presented in Table 1.

**Table 1:** Inclusion and exclusion criteria for study selection

Criteria	Inclusion	Exclusion
Population	Medical doctors learning to perform CS. (e.g., medical interns, medical officers, obstetric trainees, humanitarian surgeons, family physicians)	Studies focusing on training experienced obstetric surgeons, or exclusively on non-physicians providers performing CS independently.
Concept	Studies describing, evaluating or reporting on educational strategies in CS, including the full procedure or specific aspects of CS. Studies focusing exclusively on CS or where CS training was part of a broader training program. Any data source describing and educational approach or concept related to CS including assessment strategies and description of learning or assessment of training practices.	Studies where there is no CS training component or where the reporting exclusively on non-educational aspects of CS.
Context	No geographical restrictions were applied.	
Data sources	Research studies, descriptive studies, publications describing a training strategy or approach, conference proceedings, abstracts as well as editorial letters were considered, where the full text was available.	Review articles were excluded (though references were screened). Studies where no full text could be obtained through all reasonable means.
Language	English or where an English translation could be obtained.	Non-English studies where no feasible translation was available.

Data sources were eligible if they were published in English or if an English translation was available. Non-English literature was screened, and translations were sought where appropriate. Studies were excluded only if full-text access could not be obtained despite reasonable efforts.

### Information sources

The following databases were searched: PubMed/Medline, Epistemonikos, EMBASE, ScienceDirect, Web of Science/Scopus, and ERIC/EBSCHOST.

### Search strategy

Two investigators (LdW and SG), with support from the Stellenbosch University Faculty of Medicine and Health Sciences library services, conducted database searches covering literature from 2001 to June 2025. A combination of Medical Subject Headings (MeSH terms) and relevant keywords was used. Search results were imported into Covidence for further title and abstract screening [19].

### Data items

Search terms and combinations were tailored to each database, with the core keywords ‘Caesarean Section’ and ‘Education’ forming the basis of the strategy. Additional relevant articles were identified through the references of the identified articles and expert recommendations. The complete search strategies are detailed in Supplementary Table S2. Table 2 presents the search strategy for PubMed/Medline, as an example.

**Table 2:** Search Term Example PubMed/Medline

PubMed- Medline	Search: (caesarean section[Title/Abstract] OR cesarean section[Title/Abstract] OR c-section[Title/Abstract] OR caesarean birth[Title/Abstract] OR cesarean birth[Title/Abstract] OR cesarean[Title/Abstract] OR caesarean[Title/Abstract]) AND (teaching[Title/Abstract] OR learning[Title/Abstract] OR teach[Title/Abstract] OR learn[Title/Abstract] OR train[Title/Abstract] OR training[Title/Abstract] OR education[Title/Abstract] OR simulation[Title/Abstract] OR assessment[Title/Abstract] OR competency[Title/Abstract] OR competence[Title/Abstract]) Filters: from 2001 – June 2025

### Selection of data sources

Duplicate articles were removed manually and with Covidence software [19]. Titles and abstracts were independently screened, and inclusion required agreement between two reviewers (LdW and PvH). Disagreements were resolved through discussion or adjudication by a third reviewer (SG or KC). Full-text articles were obtained through all reasonable means, including assistance from Stellenbosch University’s library services. Manual searches and expert consultations were also conducted to identify additional sources. Review articles were excluded from the final selection, though their reference lists were screened for relevant primary studies.

### Data charting process

The final list of included sources was reviewed, and data were extracted and entered into a Redcap database by LdW and independently verified by SG [22, 23]. Key variables captured included author details, study characteristics, and main findings, as outlined in Supplementary Table S3.

### Data synthesis

Data from each included study were coded according to key domains. Through iterative discussion and consensus within the research team, the codes were grouped into four overarching categories—teaching strategies, assessment and learning outcomes, the learning curve and resource requirements, as derived from the a priori framework informed by the literature review. Individual studies could appear in multiple categories rather than being limited to one. Each study was classified according to the World Bank’s 2022–2023 country income levels based on where the study was conducted [3]. No formal critical appraisal of the data sources was undertaken, in keeping with scoping review methodology [18].

### Synthesis of results

In addition to sorting and analysing the data into the categories described, further inductive thematic analysis identified two key areas of interest, the learning curve associated with CS and the need to evaluate studies assessing educational interventions using the Kirkpatrick four-level model [24]. The Kirkpatrick model provides a framework for assessing the effectiveness of educational interventions through four sequential levels, each influencing the next (see Table 3). Results were further synthesis according to the predefined categories and the potential applicability of each study to LMICs settings.

**Table 3:** Kirkpatrick Model for evaluating educational interventions [24]

Level 1	Reaction assesses participants’ immediate responses and feelings towards the training intervention. It evaluates whether the participants enjoyed the training and found it engaging and relevant.
Level 2	Learning measures improvements in knowledge, skills, and attitudes resulting from the training intervention.
Level 3	Behaviour assesses whether learning gains translate into meaningful behavioural changes, with effectiveness defined by improved knowledge, skills, or attitudes that influence practice.
Level 4	Results evaluate the broader impact of the intervention, particularly in medical education, by assessing improvements in patient outcomes and overall quality of care.

## Results

### Study identification and selection

**Fig. 1:**
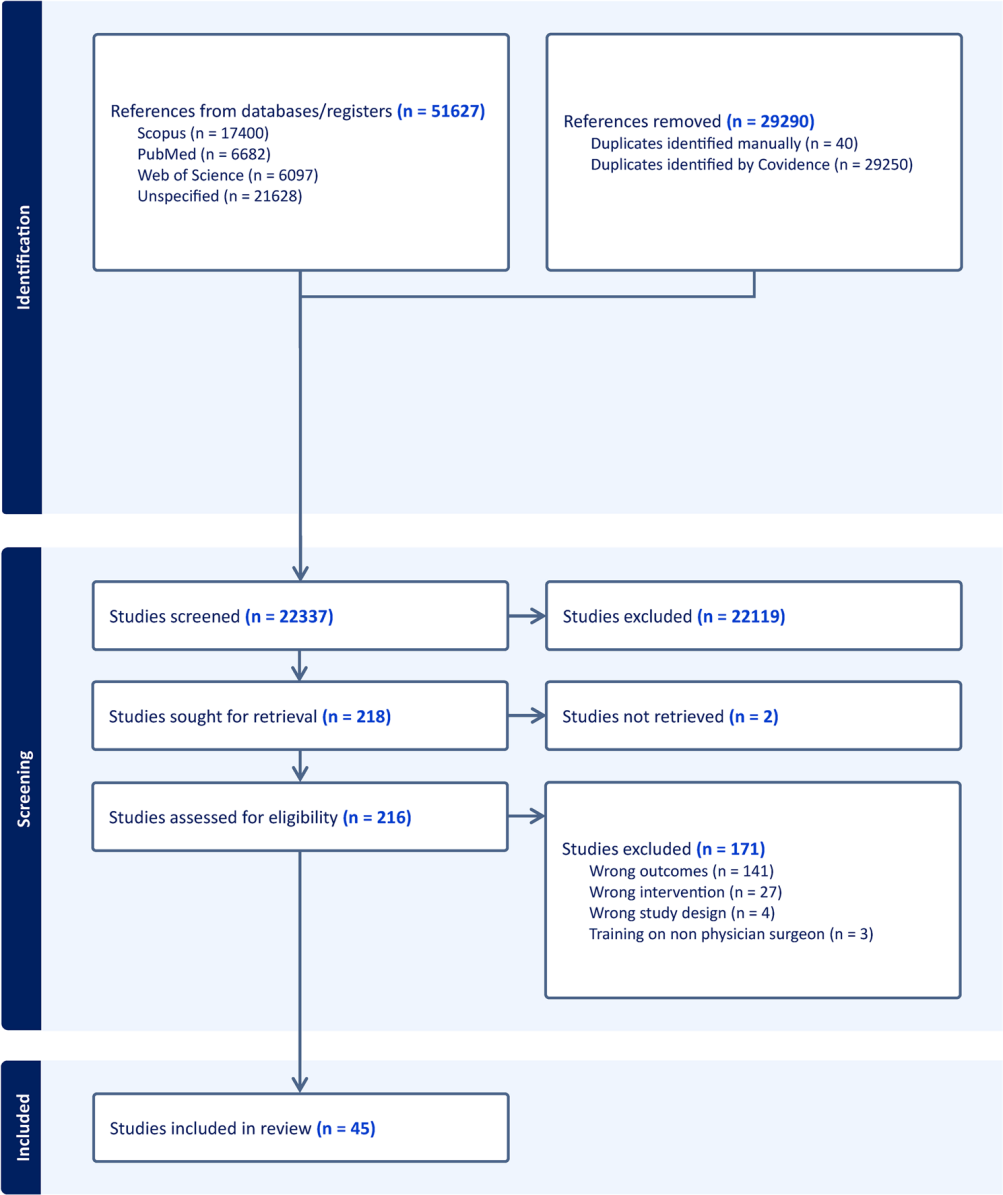
PRIMSA List of Data Selection Process

The identification and inclusion process is illustrated in Fig. 1. A total of 22,337 records underwent title and abstract screening, resulting in 216 full-text data sources being retrieved. Fourteen review articles were identified and excluded, and their reference lists yielded seven relevant data sources. A manual search added one more data source. In total, 45 data sources met the final inclusion criteria. One non-English data source was identified and an English translation was available [25].

### Study characteristics

**Fig. 2:**
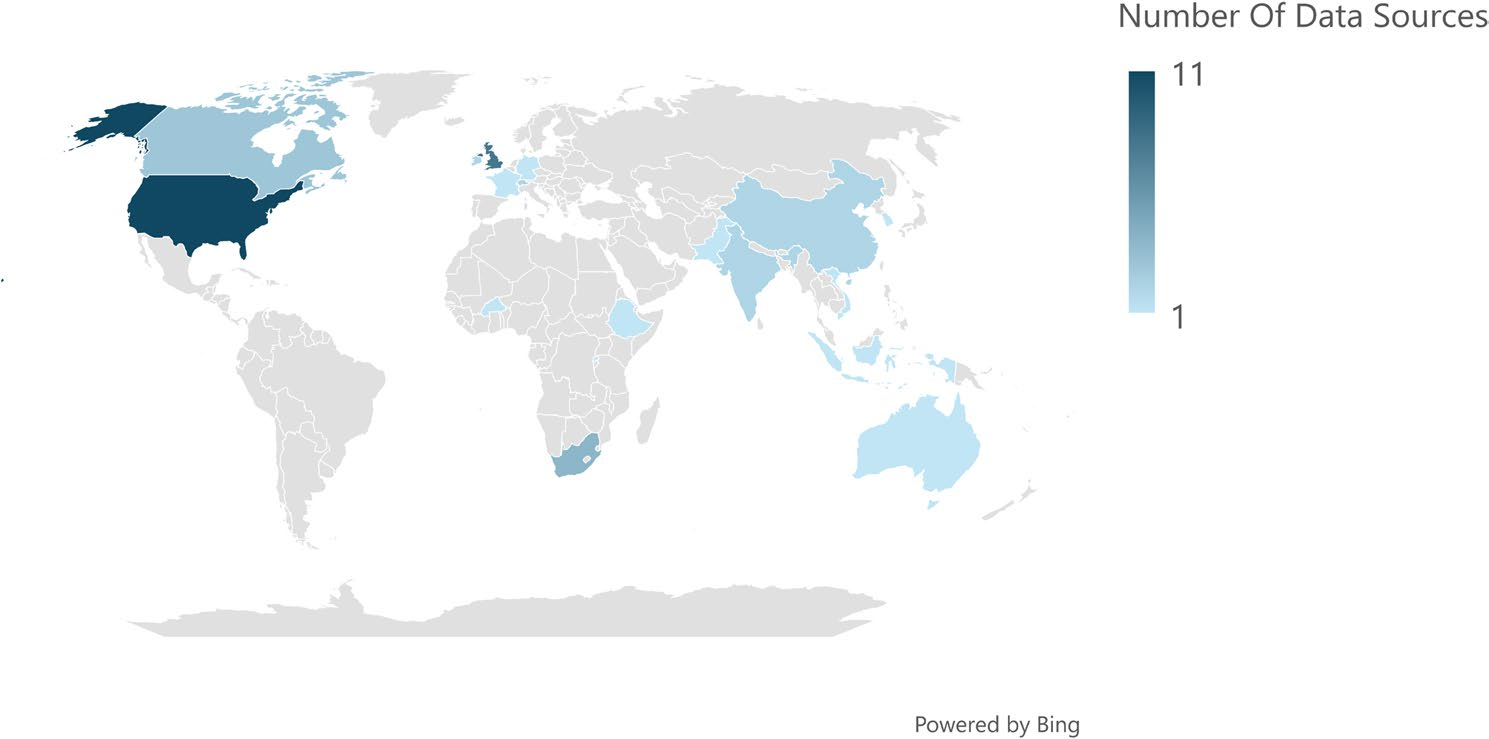
World map showing countries of data sources

Of the 45 studies, 31 (69%) originated from HICs, including 11 (24%) from the USA [26–36], 8 (17%) from the UK [37–44], and 4 (8%) from Canada [45–48], with the remainder from Ireland [48–50] Australia [49, 51] Switzerland [25, 52] Germany [53], France [54], and the Republic of Korea [55]. Fourteen data sources (31%) came from LMICs four from South Africa [56–59] and two each from China [60, 61] and India [62, 63] and one each from Indonesia [64], Pakistan [65], Vietnam [66], Ethiopia [67], Burkina Faso [68], and Rwanda [69]. Figure 2 shows the countries where the studies were conducted in on a world map. Most data sources (76%) were published within the past decade, and were journal articles (76%), followed by conference proceedings (18%).

Table 4 summarises the characteristics, research designs and educational approaches of the included studies. Most data were published between 2015 and 2025 (69%). The majority were educational interventions or before-and-after studies. Simulation-based training was the most common strategy, used in 24 studies (53%), either alone or in combination with classroom-based teaching, which was featured in 14 studies (31%).

**Table 4:** Descriptive, Research and Educational Characteristics of Included Data

Characteristic (For each study, more than one characteristic could be applicable)	Number (%) *N* = 45
Publication year	
• 2001–2014	14 (31)
• 2015–2025	31 (69)
Literature type	
• Journal article	34 (76)
• Conference proceedings (abstract/poster)	8 (18)
• Other – Short communication / commentary	3 (6)
Research Design	
• Experimental study – Randomised controlled trial	1 (2)
• Quasi-experimental study (non-randomised cohort or before-and-after study)	13 (30)
• Cross-sectional study	3 (7)
• Educational intervention study (Description with assessment of intervention)	12 (27)
• Retrospective descriptive study	4 (9)
• Mixed-methods study	2 (5)
• Non-research data source, description of the development of education tool or intervention	10 (22)
Educational Approach	
• Simulation	24 (53)
• Classroom-based teaching	14 (31)
• Practical skills course	9 (20)
• Training package/curriculum	6 (13)
• Assessment practices	7 (16)
• Cognitive task list	3 (7)
• Learning curve analysis/description	3 (7)
• Teaching videos	2 (4)
Aspects of CS Taught	
• Intraoperative aspects	45 (100)
• Pre-operative aspects	16 (37)
• Post-operative aspects	13 (30)
• Non-technical skills	8 (19)
Study Participants (where applicable)	
• Obstetric trainees	26 (60)
• Medical interns (first postgraduate year)	9 (21)
• Non-specialist clinicians	9 (21)
• Other trainees (Family medicine, general surgery)	7 (16)
• Other team members^b^ (nursing, midwife, anaesthetist)	7 (16)
• Medical students^b^	3 (7)

^a^Studies could measure more than one outcome

^b^Participated in training in addition to the novice surgeon (more than one category possible)

The included articles were further explored under the headings of teaching strategies, assessment and learning outcomes, resource requirements, and LMIC-specific studies. Table 5 outlines all the included data sources.

**Table 5:** All Included Data Sources

Title	Year Data Source Author	Country and World Bank classification	Kirk Patrick level	Training Strategy	Cost and Duration
A new course on assisted rotational birth and complex caesarean section - Mixed methods evaluation of Art & Craft [37]	2024 Journal article Jaufuraully, S	United Kingdom (UK) High Income	1	An advanced course on rotational techniques and CS at full dilation, incorporating ultrasound assessment, combined lectures with practical stations.	30-minute talk and 45-minute hands-on station Participants paid £120 each PROMPT Flex Models (limbsandthings com) ±$2,625
Creation and Evaluation of a Cesarean Section Simulator Training Program for Novice Obstetric Surgeons [39]	2023 Conference proceeding Toscano-Underwood, C	United Kingdom (UK) High Income	1,2	Described the early development of a high-fidelity, cost-effective CS training model.	Not stated
Effect of in situ simulation training for emergency caesarean section on maternal and infant outcomes [61]	2023 Journal article Wang, Y	China Upper Middle Income	1,2,4	In-situ team simulation training for CS, combined with lectures on emergency CS procedures, was evaluated.	Not stated
Immersive virtual reality simulation training for cesarean section: a randomized controlled trial [55]	2023 Journal article Kim, HJ	South Korea High Income	2	A randomized controlled trial comparing an immersive VR simulator with video and lecture-based instruction on a clinical scenario.	Not stated Single VR simulation session
Knowledge and confidence levels improvement among obstetrics residents regarding caesarean section training using video-mannequins combination [64]	2023 Journal article Kurniawati, EM	Indonesia Low Middle income	2	A quasi-experimental study compared video training, mannequin training, and their combination using pre- and post-tests.	A 13-minute video and two practice sessions on a mannequin Cost not stated
Training for managing impacted fetal head at caesarean birth: multimethod evaluation of a pilot [43]	2023 Journal article Van Der Scheer, JW	United Kingdom (UK) High Income	1,2	A training package for multidisciplinary teams managing impacted fetal head at CS was developed and evaluated The program included lectures, hands-on workshops, and simulated scenarios.	3 h PROMPT Flex Models (limbsandthings com) ±$2,625
Safe caesarean sections in South Africa: Is internship training sufficient [58]	2022 Journal article Temlett, L	South Africa Upper Middle Income	1	A study of South African internship training for safe caesarean section where the three-month obstetric rotation is intended to include the a obstetric emergency course, with each intern expected to perform 10 supervised procedures.	Four-month rotation during internship Cost not stated
Validation of a novel birth simulator for impacted fetal head at cesarean section: An observational simulation study [44]	2022 Journal article Cornthwaite, K	United Kingdom (UK) High Income	1	Multi-disciplinary professionals participated in simulated scenarios and completed questionnaires assessing validity and usefulness of the training for a novel birth simulator.	PROMPT Flex Models (limbsandthings com) was used with an estimated cost of ±$2,625, duration not stated
An Innovative Simulation Curriculum to Train General Surgery Residents and Medical Students on Four Commonly Encountered Obstetric and Gynecologic Procedures [27]	2021 Journal article Greer, JA	United States of America (USA) High Income	2	A simulation-based curriculum for general surgeons covered four gynaecological procedures—spontaneous vaginal delivery, Bartholin’s cyst incision, caesarean section, and total abdominal hysterectomy—combining lectures with simulation.	4-Hour simulation The Victoria birthing simulator was used at a cost ±$56,500
Emergency surgical obstetrics simulation training: an ex vivo low-cost model using bovine uterus and porcine bladder for haemostatic uterine brace suture techniques [40]	2021 Journal article Sinclair, A	United Kingdom (UK) High Income	1	A one-day training course on haemostatic brace suture insertion using a bovine uterus and porcine bladder demonstrated improved participant confidence.	One-day training course Cost not stated
Objective assessment of obstetrics residents’ surgical skills in caesarean: Development and evaluation of a specific rating scale [54]	2021 Journal article Berl, Q	France High Income	N/A^a^	A modified OSATS scale for caesarean section was developed and validated across varying procedural complexities.	For use in a five-year residency programme Three-minute duration Cost not stated
The development of a tool to assess the provision of essential operative obstetrical care by general surgeons in low-resource settings [47]	2021 Poster presentation Dunn, A	Canada High Income	N/A	An assessment tool was developed to support a competency-based curriculum for general surgeons providing essential operative obstetric care. Three Entrustable Professional Activities were identified: caesarean section, minor gynaecological procedures, and management of postpartum complications.	Competency assessment of a three-month training program Cost not stated
Creation and Evaluation of a Cesarean Section Simulator Training Program for Novice Obstetric Surgeons [30]	2020 Journal article Foglia, LM	United States of America (USA) High Income	2	A high-fidelity, low-cost CS simulation model was developed, with detailed design instructions provided in the article simulation, accompanied by an instructional recording.	Duration 90 min USD 25 per model that could be used twice
Improving Learners’ Comfort With Cesarean Sections Through the Use of High-Fidelity, Low-Cost Simulation [34]	2020 Journal article Acosta, T	United States of America (USA) High Income	2	A CS training program targeting critical techniques and haemorrhage management compared task trainer plus didactic education to didactic education alone.	Simulators are between ±$8000 - $14 900 each Duration not stated
A teaching video for primary caesarean Sect. [45]	2019 Conference proceeding Shore, E	Canada High Income	N/A	Canadian-developed teaching video for a primary CS Video filming of a CS is combined with 3-D animation available open access at *www tvasurg com*.	Duration 15 min video Cost of video development not stated
Is surgical skills training of medical interns in Obstetrics and Gynaecology adequate preparation for community service? [56]	2019 Journal article Lubelwana, SA	South Africa Upper Middle Income	1	This study assessed medical officers’ perceptions of internship surgical training While most were satisfied, gaps remained Recommendations included stricter program regulation, increased supervision, and raising the minimum required number of caesarean sections.	Duration Four-month internship training Cost not stated
Reducing maternal deaths by skills-and-drills training in managing obstetric emergencies: A before-and-after observational study [57]	2019 Journal article Pattinson, RC	South Africa Upper Middle Income	4	The study reported improved maternal outcomes, including reduced mortality, following implementation of the ESMOE training program.	Duration Three-day course Cost not stated
Simulation of an impacted fetal head extraction during cesarean section description of the creation and evaluation of a new training program [52]	2019 Journal article Monod, C	Switzerland High Income	1,2	A training program combining theory, clinical algorithms, and simulation for managing impacted fetal head at CS was developed using PROMPT Flex Models (limbsandthings com)	PROMPT Flex Models (limbsandthings com) was used with an estimated cost of ±$2,625
Simulation-based educational package to improve delivery of the deeply impacted fetal head at caesarean Sect. [51]	2019 Short Communication Yu, M	Australia High Income	1,2	A hospital-based training program for managing deeply impacted fetal head at CS was conducted using the Desperate Debra simulator ^38^ The program included an educational video and guided individual simulator practice.	Video with individual peer instruction practice sessions Desperate Debra simulator ±$7,846
Emergent Cesarean Section in a Bandl’s Ring Patient: An Obstetrics and Gynecology Simulation Scenario [32]	2018 Journal article Gupta, R	United States of America (USA) High Income	N/A	A simulation scenario for emergency CS in a patient with Bandl’s ring was described; however, training impact was not evaluated.	One-hour theoretical training with a simulation training scenario Cost not specified
Obstetrics knowledge and skills training as a catalyst for change [59]	2018 Journal article Pattinson, RC	South Africa Upper Middle Income	2,3	Following implementation of the Essential Steps in Managing Obstetric Emergencies (ESMOE) training package, improvements were noted across the nine signal functions of emergency obstetric care, including caesarean section.	Three-day course Cost not stated
The Basic Surgical Skills Course in Sub-Saharan Africa: An Observational Study of Effectiveness [69]	2018 Journal article Fergusson, SJ	Rwanda / Democratic Republic of the Congo Low Income	2	A two-day basic surgical skills course improved surgical technique among early-stage trainees who frequently perform caesarean sections .	Two-day course Cost not stated but had HICs support
The potential value of surrogate performance markers at caesarean section for the assessment of surgical competence [42]	2018 Journal article Sheehan H	United Kingdom (UK) High Income	N/A	Procedure time and blood loss were evaluated as metrics of caesarean section competence, both demonstrating significant reduction with increasing experience, supporting their inclusion in trainee assessment.	N/A
Evaluation of a labour ward management teaching programme in obstetrics [50]	2017 Journal article O’Leary, B	Ireland High Income	1,2	A weekly after-hours labour management course for trainees, including caesarean section, led to improved confidence and knowledge .	Weekly evenings for three months Cost not stated
Faculty and Resident Perception of a Standardized Surgical Approach and Teaching Tool for Primary Cesarean Delivery [33]	2017 Poster presentation Pallister, M	United States of America (USA) High Income	1	Faculty and resident perceptions of a standardized surgical approach for CS delivery and its associated teaching tool were evaluated.	Part of the practice was evaluated after 6 months Cost not stated
Learning From Experience: Development of a Cognitive Task List to Perform a Caesarean Section in the Obese Parturient [49]	2017 Journal Article Nicholson, S	Ireland / Australia High Income	N/A	A cognitive task list for performing CS in obese parturients was developed by senior clinicians in Ireland and Australia.	Not stated
Cesarean Section Surgical Competency Operating Room Evaluation (CS-SCORE) use has a learning curve [36]	2016 Poster presentation Jain, S	United States of America (USA) High Income	1,2	The CS-Score tool for intraoperative assessment of caesarean section competence was developed and evaluated.	Not stated
Utility of a Cesarean Delivery Surgical Skills Simulation Session for Family Medicine Residents Pursuing Surgical Obstetrics Training [35]	2016 Poster presentation Kremer, T	United States of America (USA) High Income	2	A CS surgical simulation session for family medicine trainees was evaluated using global rating scales of live surgeries pre- and post-simulation.	Not stated
Assessment of a Full Dilatation Cesarean Delivery Simulator [38]	2015 Journal article Vousden, N	United Kingdom (UK) High Income	2	The development and validation of the full dilatation CS simulator, Desperate Debra, involved three delivery scenarios of increasing difficulty.	Duration not stated Desperate Debra simulator ±$7,846
Cesarean Delivery Simulation to Improve Operative Learning [29]	2015 Poster presentation McHugh, KW	United States of America (USA) High Income	2	Low-fidelity simulator and lecture showed improved knowledge of operative steps .	Single training lecture and simulation scenario per trainee Cost not stated
Learning From Experience: Development of a Cognitive Task List to Perform a Caesarean Section in the Second Stage of Labour [48]	2015 Journal article O’Brien, S	Ireland / Canada High Income	N/A	Senior clinicians from Ireland and Canada developed a cognitive task list for CS during the second stage of labour.	Duration and cost not stated
Development and evaluation of cesarean section surgical training using computer-enhanced visual learning [31]	2014 Journal article York, SL	United States of America (USA) High Income	4	A computer-enhanced visual learning program, delivered as a video-based online module for after-hours study, was used to teach CS .	Not stated
Learning from our Ethiopian colleagues: operative obstetrics for the generalist [67]	2014 Commentary Oberai, A	Ethiopia Low Income	2	A comprehensive emergency obstetric care training program for generalists in Ethiopia, comprising lectures, theatre-based teaching, and hands-on experience with 20 caesarean sections The authors proposed that a similar program could be adapted for training generalists in Canada.	Training curriculum over three-month period Cost not stated
Assessment of competence for caesarean section with global rating scale [65]	2013 Journal article Qureshi, RN	Pakistan Low Middle Income	1	A nine-point rating scale for assessing caesarean section competence was developed and applied to forty procedures in Pakistan.	Used over nine months 40–45 min observations Cost not stated
A novel approach to teaching placement of a B-lynch suture: description of technique and validation of teaching model [26]	2012 Journal article Vetere, PF	United States of America (USA) High Income	1	A teaching model utilising a flank-steak was developed for B-Lynch suture placement.	Time not specified Cost $10
Characterisation of the learning curve of caesarean Sect. [53]	2012 Journal article Soergel, P	Germany High Income	N/A	A large longitudinal study examined the caesarean section learning curve, identifying a plateau after 10–15 procedures.	N/A
Does residency training improve cognitive competence in Obstetrics and Gynaecology surgery? [46]	2012 Poster presentation Martin, MC	Canada High Income	2	Procedure-specific competency assessment tools for caesarean section, hysterectomy, and laparoscopic tubal ligation were developed and validated.	Used over a five-year speciality training program Cost not stated
Objective Structured Assessment of Technical Skill in assessing technical competence to carry out caesarean section with increasing seniority [41]	2012 Journal article Landau, A	United Kingdom (UK) High Income	1	The Objective Structured Assessment of Technical Skill (OSATS) was applied to caesarean section Trainee feedback informed tool refinement; its use declined with increasing seniority and procedural complexity.	Used over seven years of a speciality training Cost not stated
Teaching surgical skills in obstetrics using a cesarean section simulator - bringing simulation to life [62]	2010 Journal article Vellanki, VS	India Low Middle Income	2	Low-cost, mid to high-fidelity simulators improved trainees’ comfort and knowledge of CS.	Duration not stated ±$ 500 for a simulator for 25 students
Where there is no obstetrician - increasing capacity for emergency obstetric care in rural India: An evaluation of a pilot program to train general doctors [63]	2009 Journal article Evans, CL	India Low Middle Income	3	A nationwide scale-up of a comprehensive emergency obstetric training program for medical officers (MOs) in India, which included caesarean section.	Sixteen-week training program Cost not stated
Quality cesarean delivery in Ouagadougou, Burkina Faso: A comprehensive approach [68]	2008 Journal article Richard, F	Burkina Faso Low income	4	A before-and-after study in Burkina Faso evaluated a comprehensive intervention aimed at improving access to and quality of caesarean section.	Three-year comprehensive intervention Cost not stated
Cesarean Delivery Results in a Family Medicine Residency Using a Specific Training Model [28]	2006 Journal article Heider, A	United States of America (USA) High Income	4	A family medicine CS training curriculum was implemented over three years, with defined educational outcomes and stepwise participation in CS, under obstetric specialist supervision.	3-year training curriculum Cost not stated
The effect of learning curve on the outcome of caesarean Sect. [60]	2006 Journal article Fok, WY	China Upper Middle Income	N/A	A review of the first 50 caesarean sections performed by 10 trainees demonstrated improvement after 15 procedures.	N/A
Effectiveness of Lifesaving Skills Training and Improving Institutional Emergency Obstetric Care Readiness in Lam Dong, Vietnam [66]	2006 Journal article Sloan, N	Vietnam Low Middle Income	4	The effectiveness of lifesaving skills training in emergency obstetric care, which included a one-week refresher course on caesarean section, was evaluated.	One-week refresher on caesarean section One-week training on life saving skills Cost not stated
The learning curve in the context of the cesarean Sect. [25]	2003 Journal article Muller, I	Switzerland High Income	N/A	Reported that the steepest phase of the caesarean section learning curve improved after 20 procedures.	N/A

^a^N/A: Not applicable

### Teaching strategies

A diverse set of teaching strategies was described, including traditional apprenticeship and direct mentorship in the operating room. Heider et al. described supervising trainees over three years during live CS cases with real-time feedback in a stepwise process [28]. While Oberai et al. in Ethiopia described a shorter version, with generalist doctors achieving competency within four months [67]. This hands-on approach can work well in a training setting, but does not address the challenge of addressing surgical competency in less time and with less supervision.

Several studies integrated lectures, animated videos, mannequin practice and computer-enhanced visual learning to teach key steps of a CS [31, 51, 64]. York et al., tested a video-enhanced online module that residents viewed after hours and evaluated the learning curve and the clinical outcomes (but without a control group) [31]. They stated that thirty CS are needed to achieve competency. Yu et al., used video training (detail not provided) and individual guided simulator practice using a model (Desperate Debra^®^), leading to improved knowledge, skills and confidence of learners (but no clinical outcomes measured) [51]. Kurniawati et al., in Indonesia, used a locally made mannequin with a short instructional video and showed that the combination of video and mannequin training was the most effective in increasing participants’ knowledge and confidence [64]. The model itself took 8 months to produce, using latex and silicone and conforming to exact anatomical structures [64].

Vellanki & Gillellamudi described a low-cost fabric-based multi-layer model anatomical simulation; which, combined with direct practice and supervised small-group teaching led to improved comfort and knowledge [62]. Although tested on only a handful of interns, there are photographs of their model (made from cheap fabric available from craft stores) which would make it easy to replicate [62]. Sinclair et al., described using a bovine uterus and pig bladder for a one-day course in brace-suture insertion, which led to improvement in confidence [40]. Acosta et al., describe a low-cost simulator workshop where a full CS procedure is practised on the mannequin, with video recording and direct instructor feedback [34]. The simulation showed improvement in knowledge and satisfaction from the participants. In an appendix, they describe all the steps required to modify an existing low-cost CS model using supplies that can be purchased at commonly found craft stores, superstores, or online. They also developed a full curriculum, although it is not available in the supplementary materials [34].

Some studies address specific complex scenarios such as impacted fetal head extraction or CS at full-dilation (second stage of labour). Van der Scheer et al. and Cornthwaite et al., described a course for dealing with an impacted fetal head at CS [43, 44]. The package consisted of lectures and hands-on workshops with simulated scenarios. However, they used an expensive, high-fidelity impacted fetal head simulator model (PROMPT Flex Models from limbsandthings.com). Both showed significant improvement in knowledge and comfort after the training.

Sloan et al., and Wang et al., applied lifesaving skills drills and multidisciplinary ward-based simulations into clinical workflows that included CS, reporting improved detection of life-threatening obstetric conditions and reduced decision-to-delivery time, tested in real-word settings [61, 66]. These studies did not specifically evaluate CS training practices separately by included CS as part of obstetric emergency management.

Several data sources described the development of a curricula incorporating standardised checklists, cognitive task lists, and surgical protocols that map each step of the CS from skin incision, uterine entry, fetal delivery, obtaining haemostasis, and closure [33, 48, 49]. O’Brien et al., described the development of a cognitive task list by senior clinicians from Ireland and Canada for CS in the second stage of labour [48]. Nicholson et al., described a cognitive task list to perform a CS in the obese woman. The list was developed by senior clinicians in Ireland and Australia [49] Neither task list was assessed as a training tool. Pallister et al., tested faculty and resident perceptions of a standardised surgical approach for CS delivery as a teaching tool. The intervention improved teaching opportunities for trainees based on a survey but was negatively received by faculty members [33].

Additional data sources included a Canadian-developed teaching video for primary CS, published as a conference proceeding and made available via open access at www.tvasurg.com [45]. Greer et al., developed and evaluated a simulation-based training curriculum for general surgeons covering four gynaecological procedures: spontaneous vaginal delivery, Bartholin’s cyst incision, CS and total abdominal hysterectomy [27]. The four-hour course combined didactic lectures with hands-on simulation, resulting in reported improvements in participant confidence and knowledge [27]. The study did not include comparative data or clinical outcomes.

### Assessment and learning outcomes

Comparative studies, including those comparing different training methods or before-and-after designs, reported improvements in knowledge, skill, and confidence. One randomised trial compared an immersive virtual reality (VR) simulator to traditional video and lecture formats; the VR group, trained through an 11-step virtual theatre simulation, showed greater improvement in knowledge and confidence [55] Kurniawati et al., compared video training, mannequin training, and their combination, reported that integrated video and mannequin training was most effective for enhancing knowledge and confidence [64]. Similarly, pairing didactic lectures with task trainers resulted in greater knowledge gains than lectures alone [30]. Seven studies specifically targeted CS in the second stage or management of the deeply impacted fetal head [37, 38, 43, 44, 48, 51, 52]. These included the development and validation of simulators as well as the design of associated training programs. While these interventions demonstrated improvements in knowledge and confidence, none included control groups or evaluated behavioural changes or clinical outcomes.

Wang et al., incorporated lectures on emergency CS procedures and considerations. Clinical outcomes were compared between the trained and untrained groups, demonstrating a significant reduction in the decision-to-delivery interval; however, no difference was observed in the skin incision-to-delivery interval [61]. Participants in the training group reported improved comfort and self-perceived knowledge [61].

Several studies developed or adopted formative assessment tools to evaluate competence in CS training or procedures. Berl et al., Qureshi & Ali, Jain et al., and Landau et al., described structured evaluation methods, including adaptations of the Objective Structured Assessment of Technical Skills (OSATS) specifically for CS, development of a CS-SCORE evaluation tool based on operative checklists, specific CS rating scales and checklist-based tools [36, 41, 54, 65]. None of these tools were assessed for clinical improvement of outcome.

Pattinson et al. (2019, 2018) evaluated the impact of the Essential Steps in Managing Obstetric Emergencies (ESMOE) training package on the availability of basic and comprehensive emergency and neonatal care, including CS [57, 59]. Although specific details of the CS training content were not provided, the initial study reported improvements in knowledge and modest improvements in the facility functionality, including CS outcomes [59]. The subsequent six-year before-and-after study demonstrated improved maternal outcomes, notably a reduction in CS case fatality rates, particularly in high maternal mortality regions [57]. Two additional studies on obstetrics emergency care incorporating CS also reported improved clinical outcomes [66, 68]. Similarly, Oberai et al., described a training program for generalists in comprehensive emergency obstetric care in Ethiopia, which included lectures, theatre teaching, and supervised performance of twenty CS procedures [67]. After four months, two generalists were deemed competent in CS, suggesting the model’s applicability for training generalists in Canada [67].

Evans et al., published on the nationwide implementation of a comprehensive emergency obstetric training program for medical officers (MOs) in India, which included instruction on CS procedures [63]. The program’s effectiveness was evaluated through an assessment of facility performance. However, clear improvements in service delivery were not demonstrated. This limited impact was attributed to multiple systemic deficiencies, including inadequate anaesthetic support, insufficient infrastructure and equipment, suboptimal blood transfusion services, and challenges in the selection of the trainees and participating facilities. They concluded that careful selection of candidates, alongside the presence of a well-functioning health system, is essential to ensure success in such training initiatives [63]. O’Leary et al., with the labour management course described earlier, described an increased self-reported knowledge and confidence among participants [50].

No direct comparisons between training programmes were reported and no single approach was consistently identified across studies.

### The learning curve

Sheehan (2018) evaluated the value of procedure duration and intraoperative blood loss as metrics of surgical competence in CS. Both parameters demonstrated significant reductions with increasing trainee experience, supporting their inclusion in competency assessment frameworks [42]. Temlett et al., and Lubelwana et al., examined the South African internship rotation highlighting deficiencies in safe CS [56, 58]. The internship training includes a three-month obstetric rotation, which is intended to include the ESMOE training course and requires each intern to perform ten CS supervised [57, 59]. Both survey-based studies captured the perspectives of South African community service medical officers in the year following internship, consistently identifying significant concerns regarding the interns’ preparedness for surgical responsibilities [56, 58]. Recommendations emerging from these studies included the incorporation of simulation-based training, foundational surgical skills courses, and increased exposure to complex cases.

Three studies have characterised the CS learning curve. Soergel et al., conducted a large longitudinal analysis, identifying a plateau in the curve after 10 to 15 CS procedures [53]. Fok et al., examined the first 50 CS performed by ten trainees, noting improvements after 15 cases [60]. Müller et al., reported that the steepest phase of skill acquisition occurred within the first twenty CS [25]. All three studies were done in HICs during obstetric residency programs and may not be applicable to LMICs, where training often involves non-specialists with limited supervision and fewer assisting opportunities. Berl et al. (in France) assessed the relevance of documenting the learning curve and verified the scale at different CS difficulty levels [54]. Jain et al., devised a CS surgical competency operating room evaluation (CS-Score) and assessed the use of a training video with traditional training vs. traditional training alone [36]. The CS-Score tool was found useful, but a learning curve for providing feedback was noted. The video did not improve scores, although self-reported learning improved. In the study by York et al., [32]., who also viewed the learning curve and clinical outcomes of cases, the learning curve was stated to be at 30 cases, although there was no control group.

Overall, the assessment tools described exhibited reliability and validity; however, no studies provided comparative analyses between tools or correlated assessment outcomes with clinical results. Notably, in all these studies, the learning curves were assessed following an initial unspecified training period, reflecting cases requiring supervision once baseline competency was presumed achieved [60].

### Resource requirements

Comments on most of the models and their associated costs is summarised in Table 5. Some models described were high cost (e.g. Foglia et al., in a study that targeted haemorrhage management [30]). Human resource requirements and training of supervisors were also reported as a consideration and work force implications for off-site training approaches [54, 57, 70]. Basic surgical skills training was found to be both effective and valuable; however, the initiative evaluated was developed and delivered by institutions based in HICs [69]. This raised important considerations regarding the cost, sustainability, and long-term ownership of such programs. In particular, the financial implications of training modalities were highlighted, with off-site training estimated to be approximately seven times more costly than equivalent onsite programs [57].

An additional critical consideration is the selection of appropriate candidates for training. Evidence suggests that compulsory participation by individuals lacking motivation or interest is unlikely to yield effective outcomes [63].

### Risk of bias of included studies

There was no formal risk of bias done for individual studies.

## Discussion

This scoping review aimed to synthesise the existing literature on CS training and education, with a particular focus on identifying strategies applicable to LMIC contexts. Despite the global prevalence of CS, literature addressing the educational and training aspects remains limited. Moreover, the overall quality of evidence in the available literature is low, with few studies offering robust comparative data or rigorous methodological designs.

Data from LMICs on current training practices frequently highlight the learning challenges faced by junior doctors [58, 71]. These concerns align with findings from maternal death reviews, which identify suboptimal CS training as a contributing factor in maternal mortality and CS-associated obstetric haemorrhage [4, 5] While the direct relationship between CS training and clinical outcomes remains insufficiently explored, available evidence suggests that key intraoperative factors – such as blood loss and operative duration – are closely linked to the surgeon’s level of experience [25, 42, 53, 60]. Most studies indicate that the performance in these domains tends to stabilise after approximately 15–20 procedures. Nevertheless, the trajectory and timeframe required to achieve competence are not clearly described.

The apprenticeship model remains the predominant approach to surgical training [6, 9]. However, in many LMICs, there is a demand for competent professionals capable of performing safe CS after only brief periods of training. Compounding this issue is the reliance on multiple, often junior, supervisors, which undermines the continuity of mentorship and hinders progressive acquisition of surgical skills. The fragmented supervision also limits opportunities for feedback, reflection and development of a trusted student-teacher relationship – key elements in effective surgical education [72].

In response, there is growing advocacy for alternative and standardised training strategies [6]. In other surgical disciplines these typically integrate foundational theoretical instruction, delivered through lectures or video content, with practical skills a development, spanning from basic surgical skills training to the use of low-cost or virtual simulators [73, 74]. Such structured, multimodal programs have already been successfully implemented in some LMICs settings for robotic and laparoscopic surgical training, demonstrating both feasibility and educational value [73, 74].

Competence in medical education encompasses knowledge, technical skills and professional attitudes [75]. Accordingly, it is logical that learners should acquire as much procedural understanding as possible prior to entering the operating theatre. Many of the training studies included in this review emphasised the development of knowledge and skills. Surgical skill courses and obstetric emergency training programs have demonstrated feasibility and effectiveness in LMICs contexts [57, 69, 70, 76, 77]. However, as Pattinson et al., observed, of-site training can be costly - not only in terms of administrative and training resources, but also due to the need to release healthcare workers from service delivery [57]. For long-term sustainability, training models should explore more cost-effective alternatives, including work-based approaches delivered within the clinical setting.

Blended learning approaches, such as online tutorials and instructional videos, offer particular advantages in low-resource settings by enabling asynchronous teaching and learning and reducing reliance on limited faculty resources [78]. However, their effectiveness depends on learners having sufficient time, self-directed motivation, and access to adequate infrastructure [79]. Numerous open-access educational resources are already available, mitigating the need for the development of new content. Notably, the International Federation of Obstetrics and Gynaecology (FIGO) has recently published a standardised guide outlining the procedural steps for CS [80]. Incorporating these recommendations into training curricula could enhance the clarity, consistency, and effectiveness of instructional content.

The use of simulators is associated with improvements in self-reported confidence, knowledge, and technical skills; however evidence of a direct impact on clinical outcomes remains limited [29, 32, 35, 51, 55, 62]. Simulation-based training has been shown to reduce the learning curve, particularly when applied in team training scenarios or the management of infrequent but critical complications. It offers a safe, low-stress environment for repetitive practice and skills refinement. Nevertheless, the cost of high-fidelity simulators poses a significant barrier to widespread adoption in LMICs settings. Hybrid approaches, such as the integration of video instruction with mannequin-based – as successfully implemented in Indonesia - have demonstrated positive results. Similarly, simulation-based programs in India have been effective in enhancing both procedural competence and confidence [64].

The formal assessment of competence through structured rating scales offers clear advantages for both evaluation and training. Various tools have been developed to assess different levels of CS complexity, with implementation options including peer and self-assessment where appropriate [36, 41, 42, 46, 47, 54, 65]. While the assessment methods are well-described and widely applied in HICs, their use in low-resource settings remains limited - only one data source from an LMICs reported on competency-based assessments [65]. Given their low cost and minimal resource requirements, structured assessment tools, particularly self- and peer-assessment strategies, warrant consideration in LMICs, where they may offer a feasible and sustainable approach to improving training quality and accountability [54].

### Implications

Based on the reviewed data sources, several key training components should be prioritised for implementation. Comprehensive emergency obstetric training programs, such as South Africa’s ESMOE training, serve as effective models [57, 70]. The integration of video-based instruction and simulation should be emphasised, given their demonstrated benefits in enhancing trainee comfort, confidence, knowledge, and technical skill [34, 62, 64].

Simulation is particularly valuable for preparing clinicians to manage complex scenarios, including impacted fetal head and haemostatic suturing techniques [40, 44, 51, 52]. Structured supervision should be mandatory for all novice surgeons, with a recommended minimum of 15 supervised cases prior to independent practice [25, 53, 60]. Systematic observation and structured assessment of competence using validated rating scales, incorporating a combination of self-assessment, peer review, and formal evaluation, before operating independently [54, 65]. These assessment tools should be adapted to reflect local resource settings and the complexity of the cases encountered, ensuring their feasibility and relevance within the LMICs context.

As demonstrated by Oberai et al., effective training practices implemented in LMICs settings may hold significant relevance for HICs as well [67]. However, improvements in education and training alone are insufficient to translate into better patient outcomes, in the absence of broader systemic readiness. A well-functioning health system, coupled with an enabling environment – including the availability of skilled surgical teams, supportive cultural and institutional contexts, and patient-related factors – is essential to optimise the impact of such interventions [63, 66, 68].

### Strengths

The study employed a broad search strategy, resulting in the identification of many potentially relevant studies. Importantly, no studies were excluded based on geographic location, ensuring the inclusion of data from both high- and low-resource settings.

### Limitations

The available data sources were of limited quality with only one randomised trail identified. Most studies were descriptive in nature often lacking a control group. Additional limitations included small sample size and single centre designs with little clinical or long-term data. Regarding the study methods, some relevant data sources may have been missed, particularly those published in non-English languages, indexed under alternative MESH terms, or those published after completion of the search process. Publication bias may have influenced the available literature, potentially excluding studies that reported negative findings or merely described existing training practices without demonstrating measurable improvements. In studies where CS training is well established there may be less need for research potentially limiting the availability of data on effective practices. The study protocol was not registered prior to conducting the review, which is acknowledged as a limitation and may reduce reproducibility.

### Further research

Areas that appear under-researched in this review include descriptions of current training practices both in HIC and LMICs, comparisons of different training strategies, approaches to standardisation of CS, the duration of training required to reach competence, and approaches to improve teaching skills in surgeons. The perspective and experiences of the trainer, especially in LMICs where they are often still junior could also provide valuable insights in challenges in providing CS training.

## Conclusion

This scoping review found that, although the literature on CS training remains limited, there has been a notable increase in publications over the past decade. Demonstrating a direct link between training interventions and improved clinical outcomes remains challenging, given the multifaceted nature of surgical care and the dynamics of the healthcare system. Promising teaching strategies for implementation in low resource settings include blended learning approaches, team training, and low-cost simulations. There is an urgent need for affordable, sustainable training strategies for LMICs, accompanied by an evaluation framework that assesses the impact on clinical outcomes.

## Plain language definitions (Oxford Learner’s Dictionaries) [81]

**Surgeon: **A doctor who is trained to perform surgery (medical operations that involve cutting open a person’s body).

**Novice**: A person new to and inexperienced in a job or situation.

**Novice Surgeon**: A medical doctor who is new and inexperienced in performing surgery.

**Surgery**: Medical treatment of injuries or diseases that involves cutting open a person’s body and often removing or replacing some parts; the branch of medicine connected with this treatment.

**Simulation**: A situation in which a particular set of conditions is created artificially in order to study or experience something that could exist.

**Simulator**: A piece of equipment that artificially creates a particular set of conditions in order to train somebody to deal with a situation that they may experience.

**In situ simulation training**: Training in a real-world environment where the actual situation is likely to occur.

## Supplementary Information

Supplementary Material 1.

Supplementary Material 2.

Supplementary Material 3.

## Data Availability

No datasets were generated or analysed during the current study.
