# Supplementary material for: A scoping review of educational practices in caesarean section training
Source: BMC Med Educ. 2026 May 28;26:1231. doi: 10.1186/s12909-026-09495-y (PMC13435387; doi:10.1186/s12909-026-09495-y)
Supplement: Supplementary file 2 [file 12909_2026_9495_MOESM2_ESM.docx]

**Scoping Review of Educational Practices in Caesarean Section**

**Supplementary Table S2: Search Terms**

| **Database** | **Search Term** |
| --- | --- |
| **Epistemonikos** | (title:(caesarean section) OR abstract:(caesarean section)) OR (title:(cesarean section) OR abstract:(cesarean section)) OR (title:(c-section) OR abstract:(c-section)) AND (title:(teaching) OR abstract:(teaching)) OR (title:(training) OR abstract:(training)) OR (title:(education) OR abstract:(education)) OR (title:(learning) OR abstract:(learning)) OR (title:(teach) OR abstract:(teach)) OR (title:(learn) OR abstract:(learn)) OR (title:(train) OR abstract:(train)) |
|  | Full query: (title:((title:((title:(caesarean section) OR abstract:(caesarean section)) OR (title:(c-section) OR abstract:(c-section)) OR (title:(cesarean section) OR abstract:(cesarean section)) OR (title:(caesarean delivery) OR abstract:(caesarean delivery)) OR (title:(abdominal delivery) OR abstract:(abdominal delivery)) OR (title:(caesarean operation) OR abstract:(caesarean operation))) OR abstract:((title:(caesarean section) OR abstract:(caesarean section)) OR (title:(c-section) OR abstract:(c-section)) OR (title:(cesarean section) OR abstract:(cesarean section)) OR (title:(caesarean delivery) OR abstract:(caesarean delivery)) OR (title:(abdominal delivery) OR abstract:(abdominal delivery)) OR (title:(caesarean operation) OR abstract:(caesarean operation)))) AND (title:((title:(education) OR abstract:(education)) OR (title:(educational) OR abstract:(educational)) OR (title:(training) OR abstract:(training)) OR (title:(train) OR abstract:(train)) OR (title:(teach) OR abstract:(teach)) OR (title:(teaching) OR abstract:(teaching)) OR (title:(simulation) OR abstract:(simulation)) OR (title:(teaching program) OR abstract:(teaching program)) OR (title:(training program) OR abstract:(training program))) OR abstract:((title:(education) OR abstract:(education)) OR (title:(educational) OR abstract:(educational)) OR (title:(training) OR abstract:(training)) OR (title:(train) OR abstract:(train)) OR (title:(teach) OR abstract:(teach)) OR (title:(teaching) OR abstract:(teaching)) OR (title:(simulation) OR abstract:(simulation)) OR (title:(teaching program) OR abstract:(teaching program)) OR (title:(training program) OR abstract:(training program))))) OR abstract:((title:((title:(caesarean section) OR abstract:(caesarean section)) OR (title:(c-section) OR abstract:(c-section)) OR (title:(cesarean section) OR abstract:(cesarean section)) OR (title:(caesarean delivery) OR abstract:(caesarean delivery)) OR (title:(abdominal delivery) OR abstract:(abdominal delivery)) OR (title:(caesarean operation) OR abstract:(caesarean operation))) OR abstract:((title:(caesarean section) OR abstract:(caesarean section)) OR (title:(c-section) OR abstract:(c-section)) OR (title:(cesarean section) OR abstract:(cesarean section)) OR (title:(caesarean delivery) OR abstract:(caesarean delivery)) OR (title:(abdominal delivery) OR abstract:(abdominal delivery)) OR (title:(caesarean operation) OR abstract:(caesarean operation)))) AND (title:((title:(education) OR abstract:(education)) OR (title:(educational) OR abstract:(educational)) OR (title:(training) OR abstract:(training)) OR (title:(train) OR abstract:(train)) OR (title:(teach) OR abstract:(teach)) OR (title:(teaching) OR abstract:(teaching)) OR (title:(simulation) OR abstract:(simulation)) OR (title:(teaching program) OR abstract:(teaching program)) OR (title:(training program) OR abstract:(training program))) OR abstract:((title:(education) OR abstract:(education)) OR (title:(educational) OR abstract:(educational)) OR (title:(training) OR abstract:(training)) OR (title:(train) OR abstract:(train)) OR (title:(teach) OR abstract:(teach)) OR (title:(teaching) OR abstract:(teaching)) OR (title:(simulation) OR abstract:(simulation)) OR (title:(teaching program) OR abstract:(teaching program)) OR (title:(training program) OR abstract:(training program)))))) |
| **ERIC** | ( caesarean section or cesarean section or c-section or caesarean delivery or cesarean delivery or caesarean birth or cesarean birth ) AND ( teaching or learning or education or training or train or teach or learn )  ( caesarean section or cesarean section or c-section or caesarean delivery or cesarean delivery or caesarean birth or cesarean birth ) AND ( teaching or teach or education or learning or skill or simulation or training or train or assessment or competence or competency ) |
| **Science direct** | ( caesarean section or cesarean section or c-section or caesarean delivery or cesarean delivery or caesarean birth or cesarean birth ) AND ( teaching or learning or education or training or train or teach or learn ) |
| **Pubmed- medline** | Search: **(caesarean section[Title/Abstract] OR cesarean section[Title/Abstract] OR c-section[Title/Abstract] OR caesarean birth[Title/Abstract] OR cesarean birth[Title/Abstract] OR cesarean[Title/Abstract] OR caesarean[Title/Abstract]) AND (teaching[Title/Abstract] OR learning[Title/Abstract] OR teach[Title/Abstract] OR learn[Title/Abstract] OR train[Title/Abstract] OR training[Title/Abstract] OR education[Title/Abstract] OR simulation[Title/Abstract] OR assessment[Title/Abstract] OR competency[Title/Abstract] OR competence[Title/Abstract]) Filters: Jan 2001 – June 2025** |
| **Web of Science**  **6047** | 1: TI=(caesarean section or cesarean section or c-section or caesarean delivery or caesarean birth or cesarean birth or cesarean delivery) AND TI=(teaching or teach or learning or learn or education or training or train or assessment or competence or competency or skill)  OR  2: AB=(caesarean section or cesarean section or c-section or caesarean delivery or caesarean birth or cesarean birth or cesarean delivery) AND AB=(teaching or teach or learning or learn or education or training or train or assessment or competence or competency or skill)  https://www.webofscience.com/wos/woscc/summary/3e0f9db1-7354-4cd1-8442-90ea0b7960ea-e69d899f/relevance/1 |
| **SCOPUS**  **5239**  **32,161** | ( TITLE-ABS ( caesarean AND section OR cesarean AND section OR caesarean AND birth OR cesarean AND birth OR c-section OR caesarean AND delivery OR cesarean AND delivery ) AND ( teaching OR teach OR learn OR learning OR education OR training OR train OR assessment OR competence OR competency ) ) )  ( caesarean AND section OR cesarean AND section OR c-section OR caesarean AND delivery OR cesarean AND delivery OR caesarean AND birth OR cesarean AND birth ) AND ( teaching OR teach OR education OR learning OR skill OR simulation OR training OR train OR assessment OR competence OR competency ) AND PUBYEAR > 1999 AND PUBYEAR < 2025  TITLE-ABS-KEY ( ( caesarean AND section OR c-section OR cesarean AND section OR cesarean AND birth OR caesarean AND birth ) AND ( teaching OR teach OR education OR learning OR learn OR skill OR simulation OR training OR train OR assessment OR competence OR competency ) ) AND PUBYEAR > 1999 AND PUBYEAR < 2025 |
