# Supplementary material for: A scoping review of educational practices in caesarean section training
Source: BMC Med Educ. 2026 May 28;26:1231. doi: 10.1186/s12909-026-09495-y (PMC13435387; doi:10.1186/s12909-026-09495-y)
Supplement: Supplementary file 3 [file 12909_2026_9495_MOESM3_ESM.docx]

*Data Dictionary Codebook*

Scoping review on current education practices in Caesarean Section training (PID: 4399)

17/07/2025 09:22

**Instruments**

**Instrument	Form Name**

Data charting intrument CS scoping review data_charting_intrument_cs_scoping_review

| **#** | **Field Label**  ***Field Note*** |
| --- | --- |
| 1 | Record ID |
| 2 | Title |
| 3 | Full text article |
| 4 | Publication Year |
| 5 | Publication Type 1 \| Journal article; 2 \| Review article; 3 \| Conference proceding; 4 \| Poster presentation; 5 \| Dissertation; 6 \| Other |
| 6 | If publication Type Specify |
| 7 | Publication Source - Journal / Conference name |
| 8 | Lead author surname and initial |
| 9 | Lead Author email |
| 10 | Country of lead author affiliation |

| 13 | If other specify |
| --- | --- |
| 14 | World bank classification of country |
| 15 | Key word 1 |
| 16 | Key word 2 |
| 17 | Key word 3 |
| 18 | Key word 4 |
| 19 | Key word 5 |
| 20 | Aim - purpose |
| 21 | Aim - Objective |
| 22 | Primary Outcome |
| 23 | Outcome 2 |
| 24 | Outcome 3 |
| 25 | Outcome 4 |
| 26 | Method |
| 27 | Study Design |

| 9 | study_design 9 | Quality improvement study |
| --- | --- | --- |
| 10 | study_design 10 | Before and after study |
| 11 | study_design 11 | Educational intervention study |
| 12 | study_design 12 | Other |

| 1 | participants 1 | Medical student (Pre graduate) |
| --- | --- | --- |
| 2 | participants 2 | Medical intern (Postgrad year 1/2) |
| 3 | participants 3 | Medical officer (post internship, not in training) |
| 4 | participants 4 | Obstetric trainee |
| 5 | participants 5 | Family medicine / tropical medicine trainee |
| 6 | participants 6 | Humanitarian surgeon/trainee |
| 7 | participants 7 | Other |

| 1 | Phone |
| --- | --- |
| 2 | Mail |
| 3 | Working team |
| 4 | Voluntary |
| 5 | Other |

| 28 | [if_other_study_desig n]  Show the field ONLY i f:  [study_design(12)] = '1' | If other study design | text |
| --- | --- | --- | --- |
| 29 | [start_date] | Start date | text (date_dmy) |
| 30 | [end_date] | End date | text (date_dmy) |
| 31 | [funding_resources] | Funding resources | text |
| 32 | [conficts_of_interes t] | Conflicts of interest | text |
| 33 | [participants] | Participants | checkbox |
| 34 | [if_other_participant  _speci]  Show the field ONLY i f:  [participants(7)] = '1' | IF other participant specify | text |
| 35 | [participant_discript ion] | Participant discription | text |
| 36 | [inclusion_criteria] | Inclusion criteria | text |
| 37 | [exclusion_criteria] | Exclusion criteria | text |
| 38 | [method_of_participan t_reqr] | Method of participant recruitment | radio |

| 6 | Not specified |
| --- | --- |

39 [if_other_recruitment

_metho]

Show the field ONLY i f: [method_of_participan t_reqr] = '5'

40 [number_of_participan ts]
41 [education_strategy]
42 [educational_tool_app

If other recruitment method specify

Number of participants

Education strategy Educational tool / approach

text

text

text checkbox

roach]

43 [if_other_education_s trateg]

Show the field ONLY i f: [educational_tool_appr oach(10)] = '1' and [ed ucational_tool_approac h(12)] = '1'

If other education strategy specify

1

2

3

4

5

6

7

8

9

10

11

12

text

educational_tool_approach	1

educational_tool_approach	2

educational_tool_approach	3

educational_tool_approach	4

educational_tool_approach	5

educational_tool_approach	6

educational_tool_approach	7

educational_tool_approach	8

educational_tool_approach	9

educational_tool_approach	10

educational_tool_approach	11

educational_tool_approach	12

Simu high high

Simu high low c

Simu low fi

Simu case

Team train

Class base train lectu

Onlin train cour

Pract train cour

Blen learn pack

Read prep cont

Curri Othe

44 [details_about_educat ional]

Details about educational tool used

notes

| 45 | [aspect_of_caesarean_ | Aspect of Caesarean Section Taught |
| --- | --- | --- |
|  | sectio] |  |
|  |  |  |
|  |  |  |
|  |  |  |
|  |  |  |
|  |  |  |
|  |  |  |
| 46 | [if_other_aspect_spec ify]  Show the field ONLY i f: [aspect_of_caesarean_ sectio(7)] = '1' | If other aspect specify |
| 47 | [details_about_apsect  _of_cs] | Details about aspect of CSection taught |
| 48 | [estimate_cost_of_int ervent] | Estimate cost of intervention in dollar up front |
| 49 | [estimated_cost_per_e vent_i] | Estimated cost per event in US Dollar |
| 50 | [estimate_number_of_p ersons] | Estimate number of persons trained per intervention |
| 51 | [estimate_duration_of  _educa] | Estimate duration of educational strategy in minutes |
| 52 | [outcome_measured] | Outcome measured |
|  |  |  |
|  |  |  |
|  |  |  |
|  |  |  |
|  |  |  |
|  |  |  |

c

c e

e

53 [if_other_outcome_spe cify]

Show the field ONLY i f: [outcome_measured (9)] = '1'

If other outcome specify

7

8

9

text

outcome_measured	7

outcome_measured	8

outcome_measured	9

techinical skills

Improvement in attitudes

Improved comfort with CS

Other

54 [details_one_outcome_ measur]
55 [assessment_method_us

Details one outcome measured

Assessment method used for reviewing

notes

checkbox

ed_for]

56 [if_other_method_for_ measur]

Show the field ONLY i f: [assessment_method_ used_for(7)] = '1'

improvement / outcome

If other method for measurement specify

1

2

3

4

5

6

7

text

assessment_method_used_for	1

assessment_method_used_for	2

assessment_method_used_for	3

assessment_method_used_for	4

assessment_method_used_for	5

assessment_method_used_for	6

assessment_method_used_for	7

Surv Test Inte OSA

Dire obs with she

Sup feed

Oth

57 [assessment_method_de tail]
58 [feedback_method]
59 [reported_benefit]
60 [reported_limitation s]
61 [key_finding]
62 [recommendations]
63 [sustainability_and_c ontinu]
64 [notes]
65 [data_charting_intrum ent_cs_scoping_review

_complete]

Assessment method detail

Feedback method Reported benefit Reported limitations

Key finding Recommendations

Sustainability and continuation plans

Notes

Section Header: *Form Status*

Complete?

notes

notes notes notes

notes notes notes

notes dropdown
